# Fatty Acids Profile and Consumers’ Preferences of Pecorino Cheese Manufactured from Milk of Sheep Supplemented with Flaxseed and *Ascophyllum nodosum*

**DOI:** 10.3390/foods13142165

**Published:** 2024-07-09

**Authors:** Antonella Santillo, Maria Giovanna Ciliberti, Mariangela Caroprese, Agostino Sevi, Marzia Albenzio

**Affiliations:** Department of Agriculture, Food, Natural Resources, and Engineering (DAFNE), University of Foggia, Via Napoli, 25, 71122 Foggia, Italy

**Keywords:** dairy products, small ruminants, fatty acids, human nutrition

## Abstract

The impact of flaxseed and *Ascophyllum nodosum* supplementation in ewes during the summer season on the fatty acid and sensory profile and consumer preference for cheese was evaluated. Comisana ewes (n = 32) were divided into four groups: a control (CON) group fed (30 days) with pelleted concentrate, a flaxseed (FS) group fed with whole flaxseed supplementation (250 g/ewe per day), an *A. nodosum* (AN) group fed with 5% of *A. nodosum* (into 1 kg/ewe of pelleted concentrate), and an FS + AN group fed with a combination of algae and flaxseed. Pecorino cheeses were analysed after 1 day (curd) and after 45 days (cheese) of ripening. Curd from the FS and FS + AN groups registered higher contents of MUFA, n-3, and n-3/n-6, and lower levels of atherogenic and thrombogenic indexes than curd from the CON and AN groups, as well as a higher content of C18:3n-3, C18:2t9t12, and CLA9c11t, and n-3 and n-3/n-6 fatty acids. Consumers attributed the lowest scores for appearance attributes to AN Pecorino cheese; while Pecorino cheese from FS and FS + AN was judged to have a high-strength flavour attribute and a low rancid, mouldy, and piquant flavour, in comparison with cheese from AN. Flaxseed supplementation could be an effective strategy to improve the nutritional quality of the lipid fraction of cheese without having a detrimental impact on its sensory attributes, especially during the summer season.

## 1. Introduction

Pecorino cheese is an Italian cheese made from ewe milk traditionally produced in Central and Southern Italy, contributing to the employment and income in the dairy sheep industry. Particularly, in Italy, several types of typical and traditional Pecorino cheeses are produced which are characterised by a short ripening time and semi-hard consistency [1], are made from raw, thermised or pasteurised milk [2], and are widely consumed as table cheese. However, very few studies describe the impact of different nutritional plans on Pecorino cheese quality and sensory attributes. In the last decades, there has been a re-discovery of natural and historical cheeses by the postmodern consumer which evaluates positively the place of origin by influencing their purchasing decisions towards a willingness to pay premium prices for traditional products [3]. Milk and dairy products are important components of the diet, playing an essential role in meeting nutritional requirements; in particular, cheese is rich in essential nutrients including fat, fatty acids, proteins, peptides, amino acids, vitamins, and minerals. However, cheese also contains relatively high levels of saturated fatty acids (SFAs) which are commonly perceived as negatively impacting the healthfulness of the diet and are associated with increased markers of cardiovascular risk and metabolic syndrome [4]. Dairy sheep farming systems in Mediterranean countries are based mainly on pasture, which is one of the major factors contributing to the enrichment of milk in beneficial fatty acids, especially conjugated linoleic acid (CLA). However, quantitative, and qualitative availability of pasture is greatly influenced by seasonality, and it is characterised by scarcity and poor quality during the summer season, in which dairy ewes are usually in late lactation. At this time of the year, dietary interventions based on different oils and oilseeds supplementation have been demonstrated to be beneficial for improving the fatty acid profile of milk [5]. Moreover, the manipulation of sheep diet with different plant oils has improved the fatty acid profile of cheese by raising the content of n-3 polyunsaturated fatty acid (PUFA) and CLA [6] and reducing the proportion of SFAs, thereby achieving a profile consistent with consumer perception and health recommendation. A limited number of experiments have been conducted on the evaluation of fat dietary supplementation during the summer season on the composition and quality of sheep dairy products, particularly, no previous studies have assessed the role of *A. nodosum* supplementation on dairy sheep products. Moreover, the novelty of the present study was the dietary intervention of sheep with *A. nodosum* supplementation and its combination with flaxseed, based on the hypothesis that PUFA supplementation during the summer season would improve the fatty acid profile of Pecorino-type cheese towards one with health-promoting features for human consumption. Therefore, the primary objective was to assess the effect of supplementation of ewes’ diet with flaxseed, *Ascophyllum nodosum*, and their combination on the fatty acid profile of Pecorino curd and Pecorino cheese after 45 days of ripening. A further objective was to study the sensory profile and the consumer’s preference for ripened Pecorino cheese. 

## 2. Materials and Methods

### 2.1. Animals and Experimental Design

The experiment involved 32 late-lactating Comisana ewes and lasted for 5 weeks during the summer season on a dairy farm located in Foggia (Apulia region, Italy). The first 7 days of the trial were considered an adaptation period to the experimental diets. Ewes were balanced for days in milk (200 ± 2, DIM), milk yield (271 ± 8.42 g/d), body weight (55.15 ± 1.08 kg, BW), and body condition score (2.53 ± 0.1, BCS), and were divided into four experimental groups that were individually fed twice daily. The control group (CON, n = 8) was fed with 1 kg/ewe/d of pellet concentrate (Mangimificio Molino Gallo, Potenza, Italy); the FS group (n = 8) received a supplementation of 250 g/ewe/d of whole flaxseed (Lin Tech. Tecnozoo srl, Torreselle di Piombino Dese, Italy) which was substituted to the same amount of pellet concentrate; the AN group (n = 8) received supplementation of 5% *A. nodosum* directly incorporated into pellet concentrate (Tasco); and the FS + AN group (n = 8) received supplementation with 250 g/ewe/d of flaxseed and 750 g/ewe/d of pelleted concentrate with 5% *A. nodosum* incorporated in it. The experimental groups also individually received 1.8 kg/ewe/d of oat hay with water offered ad libitum. The EU Directive 2010/63/EU guidelines [7] on the protection of animals used for experimental and other scientific purposes were followed. Animals were carefully examined by veterinarians throughout the trial to monitor their health condition. Dry matter intake (DMI) was determined by weighing the refusals four times per day (0800, 1200, 1600, and 2000 h). No differences were found for DMI among groups, being 2.62 ± 0.04 kg/ewe/d (mean value ± SEM). Experimental diets were analysed for the fatty acid composition [8]. In brief, the flaxseed supplement was characterised by a total of 53.21% of C18:3n-3 (alpha-linolenic acid, ALA), while the *A. nodosum* supplement by a total of 37.03% C18:1 cis-9 (oleic acid) and 5.03% C20:5n3 (Eicosapentaenoic acid, EPA) calculated on fatty acids. 

### 2.2. Sampling and Chemical Analyses of Milk and Pecorino Cheese

**Figure 1:**
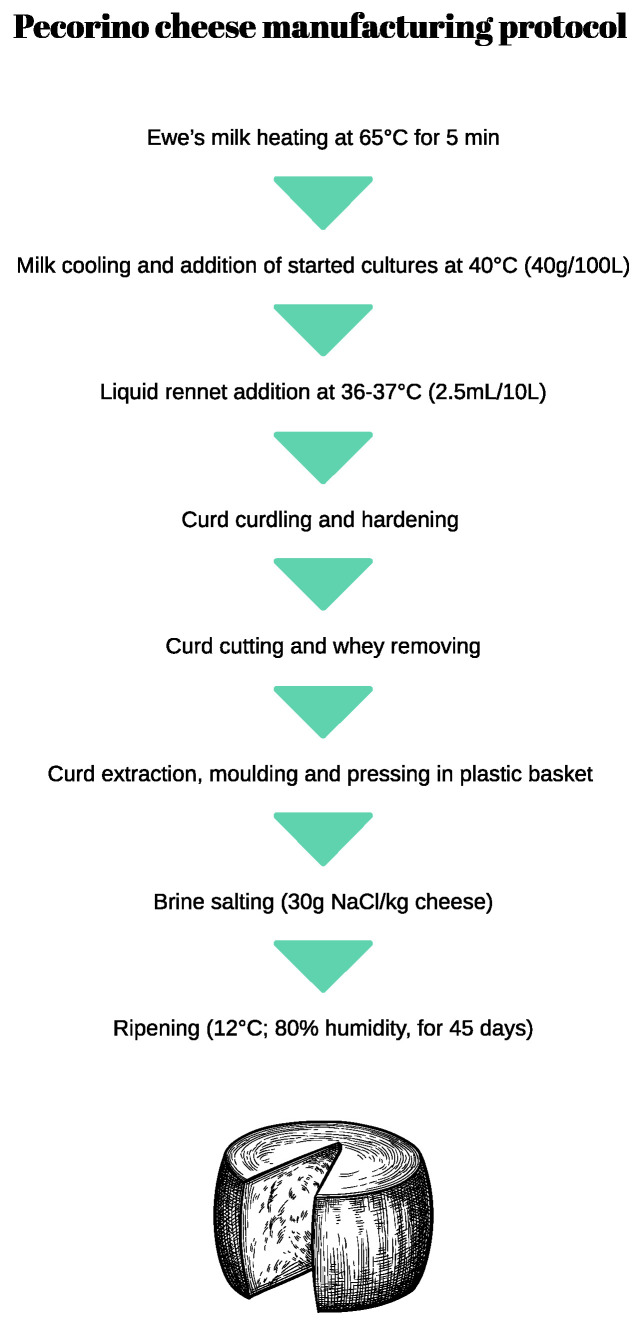
Pecorino cheese manufacturing protocol, adapted from [10].

At the end of the dietary treatments, ewes’ bulk milk from five consecutive milkings (morning and afternoon milking from 2.5 days) was collected to manufacture Pecorino cheese from each experimental group. Two cheese-making trials were performed. One fresh milk aliquot from each experimental group was collected for chemical composition determination, in terms of pH (GLP 21 Crison, Barcelona, Spain), fat, total protein, lactose, and casein content, by using MilkoScanTM FT120 (Foss Electric. DK-3400 Hillerød, Denmark) according to the International Dairy Federation standard [9]. Moreover, the evaluation of somatic cell count (SCC) by a Foss Electric Fossomatic 90 cell counter, and the renneting milk characteristics, (clotting time, rate of clot formation, and clot firmness after 30 min) using a Foss Electric formagraph, were performed. The traditional protocol of Pecorino cheese-making is shown in Figure 1. 

The chemical composition of Pecorino curd (1 day of ripening) and Pecorino cheese (45 days of ripening) was determined in terms of pH, dry matter content, moisture, and NaCl. The total nitrogen (TN), non-casein nitrogen (NCN), and non-protein nitrogen (NPN) levels were determined using the standard Kjeldahl method procedures [11]. The casein nitrogen (CN) content in the curd and cheese samples was calculated by the formula CN = (TN − NCN) × 6.38 (conversion factor), the whey protein (WP) content by the formula WP = (NCN − NPN) × 6.38 (conversion factor), and the fat content by using the Soxhlet method.

### 2.3. Fatty Acids Profile of Pecorino Curd and Cheese

Extraction of fatty acids from Pecorino curd and cheese samples was performed following the O’Fallon et al. [8] procedure. In brief, 1.0 g of cheese sample was placed into a screw-capped Pyrex tube (16 × 125 mm) and treated with C13:0 internal standard (0.5 mg of C13:0/mL of methanol), 0.7 mL of 10 N KOH, and 5.3 mL of methanol. The mixture was incubated for 1.5 h at 55 °C in a water bath and shaken every 20 min. After cooling the tube to below room temperature, 0.58 mL of H_2_SO_4_ (24 N) was added and the sample was mixed by inversion and incubated for 1.5 h at 55 °C in a water bath and shaken every 20 min. Subsequent to the cooling step, which was performed in a cold tap water bath, 3 mL of hexane was added to the tube and it was vortexed for 5 min. The fatty acid methyl ester (FAME) was collected in the hexane layer obtained after centrifugation at 500× *g* for 5 min and then placed into a GC vial and stored at −20 °C until capillary gas chromatography (CG) analysis was performed. Specifically, a capillary column (Agilent Technologies Inc. Santa Clara, CA, HP-88, 100 m × 0.25 mm × 0.20 μm) installed on an Agilent Technologies 6890N GC equipped with a flame-ionisation detector and a split injection was used. The starting oven temperature was 70 °C (held for 4 min), it was increased to 175 °C (rate of 13 °C min^−1^, held for 27 min), and finally raised to 215 °C (rate of 4 °C min^−1^, held for 45 min). The carrier gas was represented by helium with a column head pressure of 175 kPa. The temperature of both the injector and the detector was 250 °C, and the split ratio was set at 20:1. Fatty acids were identified by comparing the retention times of the fatty acids of the samples with those of the fatty acid methyl standards (FIM-FAME-7-Mix, Matreya LLC, Pleasant Gap, PA, USA) and C18:1 trans-11, C18:2 cis-9,trans-11, C18:2 cis-9,cis-11, C18:2 trans-9,trans-11, and C18:2 trans-10,cis-12 (Matreya LLC, State College, PA, USA). The peak areas were quantified using Agilent Chemstation software (B.04.03). Short-chain fatty acids (SCFAs) represent the sum of C4:0, C6:0, C8:0, C10:0, and C12:0. Medium-chain fatty acids (MCFAs) represent the sum of C14:0, C14:1c, C15:0, C16:0, C16:1c, C17:0, and C17:1c. Long-chain fatty acids (LCFAs) represent the sum of C18:0, C18:1t11, C18:1c6, C18:1c9, C18:2t9t12, C19:1t10, C19:1t7, C18:2c9c12, C20:0, C18:3n3, CLA9c11t + C20:1c11, CLA 10t,12c, C22:0, C20:4n6, C20:5n3, and C22:5n3. Atherogenic and thrombogenic indexes were calculated using the Ulbricht and Southgate [12] formulas as follows: atherogenic index (AI) = (C12:0 + 4 × C14:0 + C16:0)/[Σ MUFA + Σ PUFA(n-6) and (n-3)]; thrombogenic index (TI) = (C14:0 + C16:0 + C18:0)/[0.5 × Σ MUFA + 0.5 × Σ PUFA(n-6) + 3 × Σ PUFA(n-3) + (n-3)/(n-6)].

### 2.4. Descriptive Sensory Analysis and Consumer Test

Staff and students of the DAFNE Department of the University of Foggia were recruited for Pecorino cheese (45 days of ripening) sensory analysis. Subject information and consent forms are reported in the Supplementary Materials (Figure S1). Firstly, consumers were asked to fill out a questionnaire (Figure S2) which included information about age, sex, and the frequency of consumption of Pecorino cheese. The enrolled panel consisted of a total of 58 consumers, characterised by men and women (46 and 54%, respectively), aged 21–43, who consumed cheese regularly (frequency of consumption of at least once a week). The panellists were trained for sensory evaluation procedures and shared a specific vocabulary describing the definitions of cheese attributes. Table 1 outlines the vocabulary used for cheese appearance, colour, odour, and flavour attribute characterisation. 

Pecorino cheeses that were ripened for 45 days were allowed to remain at room temperature (22 °C) for 1 h prior to the panel consumer test to achieve an optimum condition for sensory evaluation and obtain homogeneous cuts. The consumer test procedure was previously described in [13]. Briefly, each cheese sample was assigned a random number, and slices of 1.5 × 1.5 × 1.5 cm from all four experimental groups were randomly offered to the panellists. After each cheese tasting, the consumers were invited to take a small piece of unsalted crispy bread and drink a small quantity of water. Panellists used a 10-point intensity scale to sign the cheeses’ perception. For the acceptance rating test, panellists were requested to express their overall liking on a 10-point hedonic scale from 0 (dislike extremely) to 10 (like extremely), with a neither like nor dislike neutral centre point. The order of cheese presentation between each consumer test session was performed to minimise any carryover effects [15]. At the end of the sessions, panellists were invited to express their preferred cheese among the ones tested.

### 2.5. Statistical Analysis

Data on the chemical composition of milk and the fatty acids profile of both the Pecorino curd and cheese were tested to determine a normal distribution. The differences between experimental groups were determined by ordinary one-way analysis of variance (ANOVA) with the Least Square Difference (LSD) post-hoc test for multiple pairwise comparisons. A value of *p* < 0.10 was considered as a tendency. The ability of the descriptive vocabulary to discriminate among cheeses was tested using one-way ANOVA and LSD for multiple pairwise comparison tests of the panel mean scores for each cheese. Principal component analysis was applied to a matrix of 16 sensory attributes (Appearance: chalky, uniformity, and grainy, Colour: colour uniformity, mottling, and intensity; Odour: strength, acidic, rancid; Flavour: strength, salty, acidic, piquant, bitter, sweet, mouldy, and rancid) using the PRINCOMP procedure of SAS, then the main significant two principal components were analysed using factorial analysis. All analyses were carried out in the statistical software SAS version 9.4, accessed through SAS University Edition [16].

## 3. Results and Discussion

### 3.1. Milk Chemical Composition

Recently, there has been a heightened interest in dairy production towards the enhancement of its human health-promoting properties, especially regarding the quality of the fat fraction which can be modified by proper fat dietary interventions. Notably, the response of dairy animals to fat supplementation may be related to fat source, level of supplementation, and stage of lactation [17]. In the present study, different sources of PUFA were added to the diet of sheep in late lactation during the summer season with the aim of studying their effect on milk and Pecorino cheese quality, with a focus on the fatty acids composition, sensory profile, and consumers’ preference of Pecorino cheese. The effect of FS, AN, and the combination of FS and AN on bulk-milk composition used for Pecorino cheese-making is presented in Table 2. 

No significant effect was registered in terms of milk yield (356.75 g/ewe/d ± 20.27) among experimental groups. Bulk milk significantly differed in fat (*p* = 0.03), with the AN and FS + AN groups showing higher fat content than the CON and FS groups. It was reported that the effects of marine algae supplementation in ewes’ feeding are complex and not univocal, mainly due to different basal diet compositions and dosages. Accordingly, previous results on the milk fat content in ewes demonstrated its depression [18], no change [19], or increase [20]. In the present paper, AN alone or in combination with FS seemed to sustain the fat content, probably due to the low level of CLAt10c12 found which is responsible for milk fat depression syndrome [6]. Additionally, the literature on the effect of whole flaxseed on milk fat content is controversial; however, according to the results of the present study, most of the experimental trials on the administration of whole flaxseed report no effect on ewe milk fat content [21]. Protein content in milk tended to be higher (*p* < 0.10) in the AN and FS + AN groups than in the CON one, while casein content increased in all the supplemented groups (*p* < 0.018). The positive effect on milk protein secretion may be ascribed to the phlorotannins yielded by *A. nodosum* supplementation; such compounds are able to make complexes with proteins and carbohydrates thus reducing dietary protein degradation in the rumen environment and allowing the escaped amino acids to support milk protein synthesis in the mammary gland [22]. 

Moreover, in cows supplemented with *A. nodosum*, an improvement in macromineral content in milk, in particular, P and Ca, was observed due to the high mineral content supplied by seaweed [23]. Sheep milk contains higher Ca, P, and Mg levels than other common milk sources; these minerals are largely associated with the colloidal casein micelles [24]. Based on the previous statements, AN supplementation may contribute to improved Ca and P availability in the mammary gland, in turn enhancing the efficiency of casein micelle synthesis. Concerning whole flaxseed supplementation, it is widely reported that there is a suppressive effect of dietary fat on milk protein synthesis due to the reduced availability of amino acids to the mammary gland [21]. It was previously reported that flaxseed supplementation is able to sustain the immune response during heat stress exposition, mediated by C18:3 n-3 content [25]. Therefore, the increased level of casein observed in the FS group compared to the CON group could be an outcome of the improved health status of the late lactating ewes sustaining the secretory pattern of the mammary gland during the summer season. Finally, no significant differences were registered among experimental groups in terms of the pH, somatic cell count, and renneting properties of the bulk milk destined for Pecorino cheese-making.

### 3.2. Pecorino Curd Chemical and Fatty Acids Composition

The gross composition of Pecorino curds was not different across the experimental groups, denoting that the cheese-making technology of traditional cheese was able to standardise curd gross composition. Mean composition was 60.87 ± 0.6% (mean value ± SEM), 41.55 ± 1.38%, and 20.88 ± 1.15% for moisture, fat, and protein, respectively. The fatty acid composition of Pecorino curds obtained from the milk of ewes supplemented with different sources of PUFA is reported in Table 3. 

Different sources of dietary PUFAs affected the fatty acid profile of cheese curd differently. The AN curd was characterised by the highest concentration of fatty acids, from C6:0 to C14:0. The dietary intervention with FS and FS + AN supplementation significantly decreased the level of C16:0, which was intermediate in the AN group and showed the highest values in the CON group. Both the levels of C18:0 and C18:1c9 were highest in the FS and FS + AN groups. Moreover, plain flaxseed supplementation resulted in higher levels of C18:1t11, CLA 9c11t, and CLA t10c12 fatty acids than the other experimental groups. 

The fatty acid indexes of Pecorino curd are presented in Table 4. 

The SCFA level was the highest in the AN group (*p* = 0.004), and the MCFA level was higher in the AN and CON groups than the FS and FS + AN groups (*p* < 0.001). On the contrary, these last experimental groups registered higher levels of LCFAs. Moreover, the sole FS supplementation showed the lowest level of SFAs and the highest level of PUFA and n-6. The content of MUFA, n-3, and the index n-3/n-6 was higher in the FS and FS + AN groups than in the CON and AN groups. As expected, both the FS and FS + AN groups showed lower levels of AI and TI indexes than the CON and AN groups (*p* < 0.001). Within the medium chain FAs, flaxseed supplementation improved the fatty acid composition of Pecorino curd by reducing the level of C14:0 by about 19%, and C16:0 by 21%, with both fatty acids being mainly involved in human cardiovascular risk [26]. The decreased proportions of the mentioned fatty acids in cheese curd depend directly on the conditions of milk production; accordingly, in buffalo milk, a decreased proportion of medium-chain fatty acids was reported [27] due to the whole flaxseed which can alter the ruminal ability to produce fatty acids. Furthermore, the supplementation of flaxseed exerted a leading role in the enhancement of beneficial molecules for human consumption, including vaccenic acid (C18:1t11) and CLA. Interestingly, both flaxseed and the combination of flaxseed and *A. nodosum* contributed to obtaining a favourable distribution of fatty acids in terms of the degree of acidic carbon chain unsaturation, as demonstrated by the improvement of n-3, n-3/n-6, AI, and TI of the curd, indexes which are considered crucial for the prevention and management of chronic disease [28]. In accordance with our findings, sheep supplemented with flaxseed and a combination of flaxseed and *A. nodosum* resulted in reduced levels of AI and TI indexes in individual sheep milk during the summer season [5]. 

### 3.3. Pecorino Cheese Chemical and Fatty Acids Composition

As for the Pecorino curds, the chemical composition of Pecorino cheeses ripened for 45 d did not change across the experimental treatments; mean values for moisture of 36.39 ± 0.81%, for fat of 34.66 ± 2.01%, for protein of 35.31 ± 3.41%, and for casein of 30.51 ± 3.01% were found.

The fatty acid composition of Pecorino cheeses after 45 days of ripening is presented in Table 5.

Pecorino cheese produced with milk from the FS and FS + AN groups showed lower levels of C8:0 and C10:0 fatty acids than that of the AN group. The FS and FS + AN groups showed a tendency in the level of C12:0 and 14:0, which were lower than the CON and AN groups. The FS + AN group showed the highest level of C18:0, followed by the FS and CON groups (*p* < 0.001). The C18:1t11 content was the highest in the FS group (*p* = 0.008), while the contents of C18:3n3, C18:2t9t12, and CLA9c11t fatty acids were higher in the FS and FS + AN groups than in the CON and AN groups. The fatty acid indexes of Pecorino cheese are presented in Table 6. 

The SCFA levels were higher in the AN group than in the FS group; the PUFA levels were higher in the FS and FS + AN groups than in the AN group. The n-3 and n-3/n-6 levels were higher in the FS and FS + AN groups than in the CON and AN groups. No significant differences emerged in the indexes AI and TI among the experimental groups. As for curd, the occurrence of the highest levels of both C18:1t11 and CLA in the FS group is attributed to the increase in biohydrogenation activity in the rumen supported by C18:3n-3 acid, which represented about 53% of the fatty acids yielded by flaxseed supplementation. Furthermore, vaccenic acid, which showed the highest content in the Pecorino curd and cheese, plays a key role in the synthesis of CLA cis-9, trans-11 [29] via both biohydrogenation conducted by ruminal bacteria and Δ9-desaturase activity in the udder. On the contrary, *A. nodosum* is mainly rich in eicosapentaenoic acid (representing about 5.03% of fatty acid), which was not found in different levels between the experimental groups. This result could be explained by the low transfer efficiency of EPA from the diet into milk, due to its extensive biohydrogenation at the rumen level. Moreover, the limited supply of EPA to the mammary gland, and therefore to milk, was also ascribed to the competition of its use between the mammary gland and adipose tissue [30].

The supplementation of flaxseed and the combination of flaxseed and *A. nodosum* resulted in an improved content of PUFA, n-3, and n-3/n-6 ratio also in Pecorino cheese ripened for up to 45 d, compared with plain *A. nodosum* supplementation. Therefore, especially in rearing systems where pasture is not available or during the time of the year characterised by scarcity and low-quality pasture, flaxseed supplementation can represent a feasible strategy capable of enhancing the nutritional value of the fat fraction of Pecorino cheese.

### 3.4. Descriptive Sensory Analysis and Consumer Test

The sensory attributes of Pecorino cheeses produced with milk from different dietary supplementations were categorised for appearance, colour, odour, and flavour (Table 7).

Panellists scored the lowest appearance uniformity for AN Pecorino cheese (*p* = 0.035), while the grainy attribute was judged lower in the FS + AN and FS Pecorino cheeses than in the AN one (*p* = 0.018). Moreover, the perception of colour intensity showed the lowest value in the AN Pecorino cheese (*p* = 0.001). Odour attributes, in terms of strength, acidity, and rancidity were found comparable among Pecorino cheeses. As regards the flavour attributes, no significant differences emerged among experimental Pecorino cheeses, only the strength attribute tended to be lower (*p* = 0.06) in the FS cheese than in both the AN and FS + AN Pecorino cheeses.

**Figure 2:**
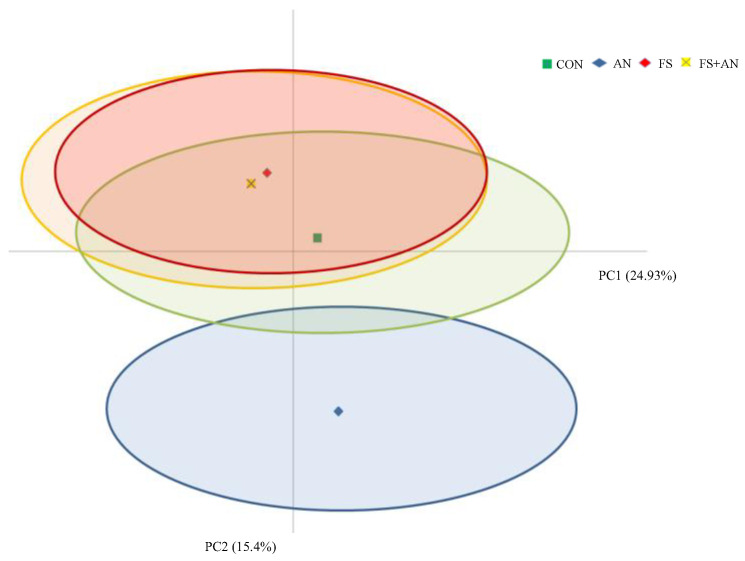
Principal Components Analysis of sensory parameters of Pecorino cheese produced from milk of sheep supplemented with flaxseed (FS, 250 g/ewe/d), *Ascophyllum nodosum* (AN, pelleted concentrate with incorporated 5% *A. nodosum*), the combination of flaxseed and *A. nodosum* (FS + AN, 250 g/ewe/d of flaxseed and 750 g/ewe/d of pelleted concentrate with incorporated 5% *A. nodosum*), or not supplemented (CON).

For the PCA results on sensory attributes, the first two principal components (PCs) were chosen as the main representatives (Table S1). In particular, PC1 accounted for 24.93% of the total variance, with the main attributes that were positively correlated being rancidity, mould, and having a piquant flavour and a rancid and acidic odour. Furthermore, the second principal component (PC2 = 15.4% of total variance) was positively characterised by the following attributes: appearance uniformity, colour intensity, and uniformity. Figure 2 shows that the FS and FS + AN Pecorino cheeses were characterised by a negative PC1 score. 

On the contrary, the AN cheese was in a well-defined zone of the plot with a negative PC2 score, categorising cheese with a lower appearance attribute. The CON cheese was located in the central part of the plot characterised by lower levels of both PC1 and PC2. The definition of a descriptive vocabulary is a key point to support the promotion and commercialisation of hard ovine cheese, especially to test the impact of managerial choices on the quality of typical and traditional dairy products. Consumers’ attribute evaluations of Pecorino cheese from *A. nodosum* supplementation were characterised by reduced appearance, uniformity, and colour intensity. These attributes were perceived negatively by the panellists, influencing the position of the AN Pecorino cheese depicted in a defined zone of PCA which described cheese with a lower appearance attribute. In Torri et al. [31], the consumers’ preferences for cheese enriched with grape skin powder (Barbera and Chardonnay) were positively influenced by the white colour, homogeneity, and elasticity of the paste. In the present experiment, the Pecorino cheese from sheep supplemented with flaxseed and the combination of flaxseed and *A. nodosum* was judged by the consumers to have a high-strength flavour attribute and a low rancid, mouldy, and piquant flavour, in comparison with the Pecorino cheese made from sheep milk supplemented with *A. nodosum*. Data on the effects of a linseed supplementation-based diet on the sensory properties of milk products are not consistent [32,33,34]. Polyunsaturated fatty acids are more prone to oxidation with the formation of unsaturated aldehydes resulting in the flavour defect referred to as oxidative rancidity; however, in cheese, the lipid oxidation is considered limited due to a low redox potential (−250 mV) [35]. Contrasting results were obtained when extruded flaxseed was integrated into the diet: production of off-flavours due to lipid oxidation was reported in cheese from cows [36] or goats [37] supplemented with flaxseed. Conversely, in sheep cheese, Caccamo et al. [38] found the absence of a detectable off-flavour in cheese matured for 40 days; this result agrees with the present research in which no differences emerged in the rancid perception among Pecorino cheese ripened for 45 days. In the present research, consumer preference was more oriented toward the conventional cheese, with the CON Pecorino cheese being preferred by 33.33% of the panel, followed by the FS group with a very close number of preferences of 28.07%, and then the FS + AN, which was preferred by 22.81%, and the AN Pecorino cheese, which was preferred by 17.54% of the panel. Consumers did not receive additional information about the different types of cheese tested, so it might be interesting to assess the impact of attributes related to the health qualities of the fatty acid profile of cheeses which cannot be ascertained from the direct tasting experience. Indeed, it was demonstrated that information about credence attributes of animal-based products is able to move the expectations towards an increase in quality perception and consumer liking [39]. A proper communication strategy would be expected to drive consumers’ preferences towards products obtained from animals under specific dietary plans that are able to sustain human health upon consumption.

## 4. Conclusions

In the present study, different sources of dietary PUFA, namely, flaxseed, *Ascophyllum nodosum*, and a combination of the two, were administered to late-lactating sheep during the summer season. *A. nodosum* supplementation to dairy ewes led to a higher fat and protein content of bulk milk destined for cheese-making. The main focus of the experimental supplementation was the study of its effect on the fatty acid profile of ovine cheese ripened for up to 45 days, with the administration of flaxseed leading to the greatest impact in this regard. Dietary flaxseed led to an increase in the level of PUFA and CLA by about 25% and 50% compared to the control, respectively, enriching the cheese matrix with molecules beneficial for human consumption. Descriptive sensory analysis was applied to characterise the sensory attributes of the cheeses, and the flaxseed-supplemented cheeses showed a sensory pattern comparable to the control cheese, whereas cheeses from the *A. nodosum* supplementation were judged to be lower in appearance uniformity and colour intensity than the control. 

Overall, data from the present study demonstrated that supplementation based on flaxseed, rather than on *A. nodosum*, could represent an effective strategy for improving the nutritional quality of the lipid fraction of cheeses, especially during seasons in which pasture is scarce and of poor quality, without exerting a negative impact on the sensory attributes of cheese. 

## Figures and Tables

**Table 1:** Descriptive vocabulary and their definition, adapted from [13,14] used by panellists to evaluate Pecorino cheese in the present study.

Attributes	Definition
Appeareance	
Chalky	Resembling chalk in appearance
Uniformity	Absence of cracks, pinholes, irregular-shaped holes
Grainy	The extent to which granular structures are formed as the sample breaks down (perceived in the second half of chewing)
Colour	
Mottling	The evenness of color shading within the cheese sample, with the most uniformly coloured cheese being free of mottling, marbling, or any other deficiencies in color
Colour Intensity	The color of cheese, ranging from pale yellow to orange, with the palest of yellow representing the start of the scale
Odour	
Strength	The overall intensity of aroma and flavor; the degree of mildness and maturity
Acidic	The smell associated with lactic and citric acids
Rancid	The smell associated with sour milk and oxidised fats, having the rank of an unpleasant aroma characteristic of oils and fats when no longer fresh
Flavour	
Strength	The overall intensity of aroma and flavor; the degree of mildness and maturity
Salty	The fundamental taste sensation of which sodium chloride is typical
Acidic	The fundamental taste sensation of which lactic and citric acids are typical
Piquant	The taste associated with an irritating or aggressive sensation perceived in the mouth or in the throat
Bitter	The fundamental taste sensation of which caffeine and quinine are typical
Sweet	The fundamental taste sensation of which sucrose is typical
Mouldy	The taste associated with moulds, usually earthy, dirty, stale, musty, and slightly sour
Rancid	The taste associated with sour milk and oxidised fats, having the rank of an unpleasant aroma or taste characteristic of oils and fats when no longer fresh

**Table 2:** Bulk-milk chemical composition for Pecorino cheese production as affected by the experimental diets.

	Experimental Diets ^1^					
Items	CON	AN	FS	FS + AN	SEM	*p*-Value
pH	6.57	6.60	6.64	6.57	0.029	NS
Fat, %	5.75 ^b^	6.78 ^a^	5.74 ^b^	6.72 ^a^	0.204	*
Protein, %	5.37 ^b^	5.85 ^a^	5.64 ^ab^	5.83 ^a^	0.084	<0.10
Lactose, %	4.26	4.64	4.56	4.66	0.074	NS
Casein, %	3.95 ^b^	4.57 ^a^	4.30 ^a^	4.55 ^a^	0.100	*
SCC ^2^, log_10_ n. cell/mL	2.81	3.10	3.04	2.89	2.31	NS
r ^3^, min	6.45	6.73	7.45	5.80	0.458	NS
a_30_^4^, mm	50.50	51.51	59.16	54.80	1.901	NS
k_20_^5^, min	1.30	1.23	1.30	1.23	0.032	NS

^a–b^ Mean values in the same row with different superscripts differ (*p* < 0.05). * *p* < 0.05, <0.10 tendence, NS = *p* > 0.10. ^1^ CON = sheep fed conventional diet; AN = sheep supplemented with 5% *Ascophyllum nodosum* directly incorporated into pellet concentrate (Tasco); FS = sheep supplemented with 250 g/ewe/d of whole flaxseed; FS + AN= sheep supplemented with 250 g/ewe/d of flaxseed and 750 g/ewe/d of pelleted concentrate with incorporated 5% *A. nodosum*. ^2^ SCC= Somatic Cell Count. ^3^ r = rennet coagulation time (min). ^4^ a_30_= curd firmness 30 min after enzyme addition (mm). ^5^ k_20_ curd firmness traits (min) [time to curd firmness of 20 mm (k_20_)].

**Table 3:** Fatty acids composition of Pecorino curd as affected by the experimental diets.

	Experimental Diets ^1^					
Item	CON	AN	FS	FS + AN	SEM	*p*-Value
FA, g/100 g of FA						
C4:0	3.38	4.28	3.58	3.99	0.233	NS
C6:0	1.69 ^b^	2.56 ^a^	1.42 ^b^	1.79 ^b^	0.144	*
C8:0	1.58 ^b^	2.54 ^a^	1.24 ^b^	1.59 ^b^	0.150	*
C10:0	4.54 ^b^	7.14 ^a^	3.47 ^b^	4.38 ^b^	0.401	**
C12:0	3.14 ^b^	4.32 ^a^	2.64 ^b^	2.98 ^b^	0.181	**
C14:0	10.52 ^a^	11.15 ^a^	8.37 ^b^	8.64 ^b^	0.327	*
C16:0	28.51 ^a^	26.04 ^b^	22.54 ^c^	21.99 ^c^	0.746	***
C16:1c	1.32 ^a^	1.25 ^a^	1.07 ^b^	0.98 ^b^	0.037	***
C18:0	8.59 ^b^	7.26 ^b^	9.71 ^ab^	10.54 ^a^	0.361	**
C18:1t11	2.11 ^c^	3.28 ^b^	6.21 ^a^	3.79 ^b^	0.411	***
C18:1c9	23.81 ^b^	20.10 ^c^	25.78 ^ab^	26.90 ^a^	0.733	***
C18:2t9t12	0.13 ^b^	0.11 ^b^	0.18 ^a^	0.13 ^b^	0.008	*
C18:2c9c12	2.85 ^a^	2.73 ^ab^	2.43 ^c^	2.55 ^bc^	0.050	**
C18:3n3	0.76 ^b^	0.98 ^b^	2.07 ^a^	2.05 ^a^	0.163	***
CLA9c11t	0.64 ^c^	0.93 ^bc^	1.93 ^a^	1.31 ^b^	0.139	***
CLAt10c12	0.04 ^c^	0.05 ^b^	0.11 ^a^	0.04 ^c^	0.007	***
C22:0	0.10 ^a^	0.04 ^b^	0.05 ^ab^	0.04 ^b^	0.009	*
C20:4n6	0.20 ^ab^	0.23 ^b^	0.19 ^a^	0.17 ^b^	0.008	<0.10
C20:5n3	0.08 ^a^	0.05 ^b^	0.07 ^ab^	0.06 ^ab^	0.005	NS
C22:5n3	0.08 ^a^	0.06 ^b^	0.09 ^a^	0.09 ^a^	0.003	**

^a–c^ Mean values in the same row with different superscripts differ (*p* < 0.05). * *p* < 0.05, ** *p* < 0.01, *** *p* < 0.001, <0.10 tendence, NS = *p* > 0.10. **^1^** CON = sheep fed conventional diet; AN = sheep supplemented with 5% *Ascophyllum nodosum* directly incorporated into pellet concentrate (Tasco); FS = sheep supplemented with 250 g/ewe/d of whole flaxseed; FS + AN = sheep supplemented with 250 g/ewe/d of flaxseed and 750 g/ewe/d of pelleted concentrate with incorporated 5% *A. nodosum*.

**Table 4:** Fatty acid indexes of Pecorino curd as affected by the experimental diets.

	Experimental Diets ^1^					
Item	CON	AN	FS	FS + AN	SEM	*p*-Value
SCFA ^2^	13.34 ^b^	20.84 ^a^	12.35 ^b^	14.74 ^b^	1.32	**
MCFA ^3^	42.45 ^a^	40.14 ^a^	33.80 ^b^	33.25 ^b^	0.75	***
LCFA ^4^	39.92 ^b^	36.70 ^b^	50.85 ^a^	49.44 ^a^	1.06	***
SFA ^5^	64.57 ^b^	67.48 ^a^	55.47 ^d^	58.15 ^c^	1.289	***
MUFA ^6^	30.30 ^b^	27.12 ^c^	37.07 ^a^	35.16 ^a^	1.059	***
PUFA ^7^	5.13 ^c^	5.41 ^c^	7.45 ^a^	6.70 ^b^	0.260	***
P/S ^8^	0.08 ^c^	0.08 ^c^	0.13 ^a^	0.12 ^b^	0.006	***
n-6	4.15 ^b^	4.29 ^b^	5.18 ^a^	4.46 ^b^	0.123	**
n-3	0.97 ^b^	1.13 ^b^	2.27 ^a^	2.24 ^a^	0.165	***
n-3/n-6	0.24 ^b^	0.26 ^b^	0.44 ^a^	0.50 ^a^	0.031	***
AI ^9^	2.08 ^b^	2.32 ^a^	1.32 ^c^	1.43 ^c^	0.113	***
TI ^10^	2.34 ^a^	2.30 ^a^	1.43 ^b^	1.53 ^b^	0.111	***

^a–c^ Mean values in the same row with different superscripts differ (*p* < 0.05). ** *p* < 0.01, *** *p* < 0.001. ^1^ CON = sheep fed conventional diet; AN = sheep supplemented with 5% *Ascophyllum nodosum* directly incorporated into pellet concentrate (Tasco); FS = sheep supplemented with 250 g/ewe/d of whole flaxseed; FS + AN = sheep supplemented with 250 g/ewe/d of flaxseed and 750 g/ewe/d of pelleted concentrate with incorporated 5% *A. nodosum*. ^2^ SCFA= Short Chain Fatty Acids. ^3^ MCFA= Medium Chain Fatty Acids. ^4^ LCFA = Long Chain Fatty Acids. ^5^ SFA = Saturated Fatty Acids. ^6^ MUFA = Monounsaturated Fatty Acids. ^7^ PUFA = Polyunsaturated Fatty Acids. ^8^ P/S = Polyunsaturated Fatty Acids/Saturated Fatty Acids. ^9^ AI = Atherogenic Index. ^10^ TI = Thrombogenic index.

**Table 5:** Fatty acid composition of Pecorino cheese as affected by the experimental diets.

	Experimental Diets ^1^					
Item	CON	AN	FS	FS + AN	SEM	*p*-Value
FA, g/100 g of FA						
C4:0	3.63	3.45	2.53	2.98	0.244	NS
C6:0	1.85 ^ab^	2.13 ^a^	1.19 ^b^	1.41 ^ab^	1.449	NS
C8:0	1.78 ^ab^	2.2 ^a^	1.18 ^b^	1.4 ^b^	0.137	*
C10:0	5.03 ^ab^	6.26 ^a^	3.47 ^c^	3.9 ^bc^	0.348	**
C12:0	3.62 ^ab^	3.93 ^a^	2.80 ^b^	2.96 ^b^	0.178	<0.10
C14:0	12.71 ^a^	10.71 ^ab^	9.49 ^b^	9.46 ^b^	0.549	<0.10
C16:0	28.11	26.75	27.96	26.64	2.458	NS
C16:1c	1.63 ^a^	1.23 ^ab^	1.26 ^ab^	1.12 ^b^	0.087	NS
C18:0	10.91 ^b^	8.13 ^c^	12.52 ^b^	14.72 ^a^	0.697	**
C18:1t11	3.36 ^b^	3.79 ^b^	9.21 ^a^	5.73 ^b^	1.05	**
C18:1c9	29.47	21.43	13.64	14.34	3.095	NS
C18:1c11	0.44	0.42	0.57	0.49	0.032	NS
C18:2t9t12	0.18 ^b^	0.17 ^b^	0.63 ^a^	0.68 ^a^	0.077	**
C18:2c9c12	3.67	2.92	3.07	3.23	0.176	NS
C18:3n3	0.79 ^b^	0.80 ^b^	1.93 ^a^	2.16 ^a^	0.182	**
CLA9c11t	0.93 ^b^	0.89 ^b^	1.97 ^a^	1.77 ^a^	0.165	*
CLAt10c12	0.05 ^b^	0.06 ^b^	0.11 ^a^	0.06 ^b^	0.007	**
C22:0	0.12 ^a^	0.07 ^b^	0.10 ^ab^	0.08 ^ab^	0.009	NS
C20:4n-6	0.21 ^a^	0.18 ^ab^	0.16 ^b^	0.17 ^ab^	0.008	NS
C20:5n-3	0.09	0.06	0.07	0.09	0.007	NS
C22:5n-3	0.09 ^ab^	0.07 ^b^	0.10^a^	0.11 ^a^	0.006	*

^a–c^ Mean values in the same row with different superscripts differ (*p* < 0.05). * *p* < 0.05, ** *p* < 0.01, <0.10 tendence, NS = *p* > 0.10. ^1^ CON = sheep fed conventional diet; AN = sheep supplemented with 5% *Ascophyllum nodosum* directly incorporated into pellet concentrate (Tasco); FS = sheep supplemented with 250 g/ewe/d of whole flaxseed; FS + AN = sheep supplemented with 250 g/ewe/d of flaxseed and 750 g/ewe/d of pelleted concentrate with incorporated 5% *A. nodosum*.

**Table 6:** Fatty acid indexes of Pecorino cheese after 45 days of ripening as affected by the experimental diets.

	Experimental Diets ^1^					
Item	CON	AN	FS	FS + AN	SEM	*p*-Value
SCFA ^2^	15.91 ^ab^	17.96 ^a^	11.18 ^b^	12.75 ^ab^	1.67	*
MCFA ^3^	30.93	40.50	41.07	39.28	3.96	NS
LCFA ^4^	53.90	43.35	54.65	51.17	3.37	NS
SFA ^5^	56.48	65.56	63.76	65.97	2.066	NS
MUFA ^6^	37.05	28.95	28.17	25.27	2.376	NS
PUFA ^7^	6.48 ^ab^	5.49 ^b^	8.59 ^a^	8.76 ^a^	0.531	*
P/S ^8^	0.12 ^ab^	0.08 ^b^	0.14 ^a^	0.13 ^a^	0.008	*
n-6	5.43	4.52	6.43	6.34	0.380	NS
n-3	1.05 ^b^	0.97 ^b^	2.16 ^a^	2.42 ^a^	0.189	***
n-3/n-6	0.21 ^b^	0.21 ^b^	0.34 ^a^	0.39 ^a^	0.023	***
AI ^9^	1.62	2.14	2.1	2.15	0.162	NS
TI ^10^	1.62	2.3	2.21	2.22	0.160	NS

^a–b^ Mean values in the same row with different superscripts differ (*p* < 0.05), * *p* < 0.05, *** *p* < 0.001, NS = *p* > 0.10. **^1^** CON = sheep fed conventional diet; AN = sheep supplemented with 5% *Ascophyllum nodosum* directly incorporated into pellet concentrate (Tasco); FS = sheep supplemented with 250 g/ewe/d of whole flaxseed; FS + AN = sheep supplemented with 250 g/ewe/d of flaxseed and 750 g/ewe/d of pelleted concentrate with incorporated 5% *A. nodosum*. ^2^ SCFA = Short Chain Fatty Acids. ^3^ MCFA = Medium Chain Fatty Acids. ^4^ LCFA = Long Chain Fatty Acids. ^5^ SFA = Saturated Fatty Acids. ^6^ MUFA = Monounsaturated Fatty Acids. ^7^ PUFA = Polyunsaturated Fatty Acids. ^8^ P/S = Polyunsaturated Fatty Acids/Saturated Fatty Acids. ^9^ AI = Atherogenic Index. ^10^ TI = Thrombogenic index.

**Table 7:** Sensory attributes assessed by panellists after the consumer test of the Pecorino cheeses as affected by the experimental diets.

	Experimental Diets ^1^					
Attributes	CON	AN	FS	FS + AN	SEM	*p*-Value
Appeareance						
Chalky	5.05	5.21	4.95	4.53	0.15	0.405
Uniformity	5.97 ^a^	5.215 ^b^	6.19 ^a^	6.19 ^a^	0.14	*
Grainy	5.19 ^ab^	5.55 ^a^	4.83 ^b^	4.24 ^b^	0.15	*
Colour						
Colour Uniformity	6.39	6.03	6.45	6.47	0.12	NS
Colour intensity	5.69 ^a^	4.53 ^b^	6.03 ^a^	5.48 ^a^	0.14	**
Odour						
Strength	5.92	5.66	5.53	5.91	0.12	NS
Acidic	3.28	3.64	3.00	3.07	0.15	NS
Rancid	2.14	2.19	2.05	2.02	0.15	NS
Flavour						
Strength	6.31 ^ab^	6.60 ^a^	5.72 ^b^	6.36 ^a^	0.12	<0.10
Salty	5.76	5.52	5.34	5.12	0.13	NS
Acidic	3.91	4.26	3.53	3.90	0.16	NS
Piquant	2.40	2.79	2.78	2.29	0.15	NS
Bitter	2.83	3.31	2.84	3.24	0.16	NS
Sweet	2.60	2.76	3.48	2.93	0.17	NS
Mould	1.41	1.55	1.17	1.21	0.13	NS
Rancid	2.19	2.24	1.62	1.86	0.16	NS

^a–b^ Mean values in the same row with different superscripts differ (*p* < 0.05), * *p* < 0.05, ** *p* < 0.01, <0.10 tendency, NS = *p* > 0.10. ^1^ CON = sheep fed conventional diet; AN = sheep supplemented with 5% *Ascophyllum nodosum* directly incorporated into pellet concentrate (Tasco); FS = sheep supplemented with 250 g/ewe/d of whole flaxseed; FS + AN = sheep supplemented with 250 g/ewe/d of flaxseed and 750 g/ewe/d of pelleted concentrate with incorporated 5% *A. nodosum*.

## Data Availability

The original contributions presented in the study are included in the article and Supplementary Material, further inquiries can be directed to the corresponding author.

## Supplementary Materials

The following supporting information can be downloaded at: https://www.mdpi.com/article/10.3390/foods13142165/s1, Figure S1: Subject information and consent form, Figure S2: Questionnaire for Pecorino Cheese consumer test, Table S1: Results of Principal component analysis on sensory analysis consumer test Pecorino cheeses from the experimental diets, showing the loadings of the first five Principal Component.
