# Supplementary material for: Fatty Acids Profile and Consumers’ Preferences of Pecorino Cheese Manufactured from Milk of Sheep Supplemented with Flaxseed and Ascophyllum nodosum
Source: Foods. 2024 Jul 9;13(14):2165. doi: 10.3390/foods13142165 (PMC11276284; doi:10.3390/foods13142165)
Supplement: Supplementary file 1 [file foods-13-02165-s001.zip › foods-3057237-supplementary.pdf]

Figure S1: Subject information and consent form

I __________________________ agree to participate to the consumer panel on Pecorino cheeses within the research project titled "Fatty acids profile and consumers' preferences of Pecorino cheese manufactured from milk of sheep supplemented with flaxseed and Ascophyllum nodosum" conducted by researchers Antonella Santillo, Maria Giovanna Ciliberti, Mariangela Caroprese, Agostino Sevi, and Marzia Albenzio who have discussed the research project with me. The purpose of this study is to identify the sensory profile and the consumers preference of ripened (45 days) Pecorino cheese obtained from sheep supplemented with flaxseed, *Ascophyllum nodosum* and their combination.

I have received, read, understood, and kept a copy of the information letter. I have had the opportunity to ask questions regarding this project research. I consent to participate in this research project and the following has been explained to me:

- The research may not be of direct benefit to me
- My participation is completely voluntary
- My right to withdraw from the study at any time without any implication to me
- What I am expecting and required to do
- The procedure of this research
- Security and confidentiality of my personal information
- I am able to request a copy of the research findings or report
- In addition, I consent to the treatment of my personal data to the University of Foggia which will process data only with the purpose of this research project,

- data will be used solely for identifying the preference on cheeses tested and for describing the information about age, sex, and the frequency of consumption of Pecorino cheese that will be presented in a paper in an anonymous way.

Figure S2: Questionnaire for Pecorino Cheese consumer test

Age:____

Sex:_____

Which is your frequency of consumption of Pecorino cheese?

- o Every day
- o At least once a week
- o Weekly
- o Monthly

Please use the following 10-point intensity scale to sign the cheeses' perception for each attribute based on the descriptive vocabulary and the definition provided. For the acceptance rating test, please express the overall liking on a 10-point hedonic scale from 0 (dislike extremely) to 10 (like extremely), and with a neither like nor dislike neutral center point. After each cheese tasting you are invited to take a small piece of unsalted crispy bread and drink a small quantity of water.

Appeareance Chalky

1	2	3	4	5	6	7	8	9	10

Appeareance Uniformity

1	2	3	4	5	6	7	8	9	10

Appeareance Grainy

1	2	3	4	5	6	7	8	9	10

Colour Mottling

1	2	3	4	5	6	7	8	9	10

Colour Intensity

1	2	3	4	5	6	7	8	9	10

Odour Strength

1	2	3	4	5	6	7	8	9	10

Odour Acidic

1	2	3	4	5	6	7	8	9	10

Odour Rancid

1	2	3	4	5	6	7	8	9	10

Flavour Strength

1	2	3	4	5	6	7	8	9	10

Flavour Salty

1	2	3	4	5	6	7	8	9	10

Flavour Acidic

1	2	3	4	5	6	7	8	9	10

Flavour Piquant

1	2	3	4	5	6	7	8	9	10

Flavour Bitter

1	2	3	4	5	6	7	8	9	10

Flavour Sweet

1	2	3	4	5	6	7	8	9	10

Flavour Moldy

1	2	3	4	5	6	7	8	9	10

Flavour Rancid

1	2	3	4	5	6	7	8	9	10

Please expresses your preferred cheese among the ones tested.

1°_______________

2°_______________

3°_______________

4°_______________

Table S1: Results of Principal component analysis on sensory analysis consumer test Pecorino cheeses from the experimental diets, showing the loadings of the first five Principal Component.

	Principal Component				
**Descriptor**	**1**	**2**	**3**	**4**	**5**
Odour_ Rancid	.789	.058	-.048	.283	-.096
Flavour_Mold	.771	-.098	.055	-.004	.025
Flavour _Rancid	.747	.024	-.024	.274	.182
Flavour _Piquant	.607	-.088	.501	-.329	-.046
Odour_Acidic	.578	.025	.135	.457	.065
Appearence_Uniformity	-.029	.861	.026	.002	.090
Colour_Uniformity	-.084	.767	.065	.193	.043
Colour_Intensity	.066	.758	.124	-.109	-.243
SA_Strenght	-.081	.149	.765	.175	.089
SA_Salty	.144	.030	.734	.113	.210
Odour_Strength	.021	.397	.485	.195	-.269
Appearence_Chalky	.174	.174	.079	.739	.052
Appearence_Grainy	.216	-.081	.337	.637	-.122
Flavour_Bitter	.488	.115	.073	.056	.636
Flavour_Sweet	.338	.210	-.288	.211	-.596
Flavour_Acidic	.475	-.121	.041	.370	.483

The most important descriptors on each PC are highlighted in red, variables which are characterized by the negative values, and in yellow, the variables characterized by the negative values.
